# BRCA Detection Rate in an Italian Cohort of Luminal Early-Onset and Triple-Negative Breast Cancer Patients without Family History: When Biology Overcomes Genealogy

**DOI:** 10.3390/cancers12051252

**Published:** 2020-05-15

**Authors:** Angela Toss, Eleonora Molinaro, Marta Venturelli, Federica Domati, Luigi Marcheselli, Simonetta Piana, Elena Barbieri, Giovanni Grandi, Claudia Piombino, Isabella Marchi, Elena Tenedini, Enrico Tagliafico, Giovanni Tazzioli, Laura Cortesi

**Affiliations:** 1 Department of Oncology and Hematology, Azienda Ospedaliero-Universitaria di Modena, 41124 Modena, Italy; ele.molinaro.89@gmail.com (E.M.); martaventurelli@msn.com (M.V.); federica.domati@unimore.it (F.D.); lmarcheselli@unimore.it (L.M.); barbieri.elena@aou.mo.it (E.B.); claudia.piombino@outlook.com (C.P.); marchi.isabella@policlinico.mo.it (I.M.); hbc@unimore.it (L.C.); 2 Department of Surgery, Medicine, Dentistry and Morphological Sciences with Transplant Surgery, Oncology and Regenerative Medicine Relevance, University of Modena and Reggio Emilia, 41124 Modena, Italy; 3 Pathology Unit, Azienda USL-IRCCS Reggio Emilia, 42123 Reggio Emilia, Italy; Simonetta.Piana@ausl.re.it; 4 Department of Obstetrics, Gynecology and Pediatrics, Obstetrics and Gynecology Unit, Azienda Ospedaliero-Universitaria di Modena, 41124 Modena, Italy; giovanni.grandi@unimore.it; 5 Department of Laboratory Medicine and Pathology, Diagnostic hematology and Clinical Genomics Unit, Modena University Hospital, 41124 Modena, Italy; tenedini.elena@gmail.com (E.T.); enrico.tagliafico@unimore.it (E.T.); 6 Center for Genome Research University of Modena and Reggio Emilia, 41124 Modena, Italy; 7 Department of Medical and Surgical Sciences, University of Modena and Reggio Emilia, 41124 Modena, Italy; giovanni.tazzioli@unimore.it; 8 Oncologic Breast Surgery Unit, Azienda Ospedaliero-Universitaria Policlinico, University Hospital of Modena, 41124 Modena, Italy

**Keywords:** BRCA, family history, genetic testing, triple-negative, early-onset, breast cancer

## Abstract

NCCN Guidelines recommend BRCA genetic testing in individuals with a probability >5% of being a carrier. Nonetheless, the cost-effectiveness of testing individuals with no tumor family history is still debated, especially when BRCA testing is offered by the national health service. Our analysis evaluated the rate of BRCA pathogenic or likely-pathogenic variants in 159 triple-negative breast cancer (TNBC) patients diagnosed ≤60 years, and 109 luminal-like breast cancer (BC) patients diagnosed ≤35 without breast and/or ovarian family histories. In TNBC patients, BRCA mutation prevalence was 22.6% (21.4% BRCA1). Mutation prevalence was 64.2% ≤30 years, 31.8% in patients aged 31–40, 16.1% for those aged 41–50 and 7.9% in 51–60 s. A total of 40% of patients with estrogen receptors (ER) 1–9% were BRCA1 carriers. BRCA detection rate in early-onset BCs was 6.4% (4.6% *BRCA2*). Mutation prevalence was 0% between 0–25 years, 9% between 26–30 years and 6% between 31–35 years. In conclusion, BRCA testing is recommended in TNBC patients diagnosed ≤60 years, regardless of family cancer history or histotype, and by using immunohistochemical staining <10% for both ER and/PR. In luminal-like early-onset BC, a lower BRCA detection rate was observed, suggesting a role for other predisposing genes along with BRCA genetic testing.

## 1. Introduction

First- and second-degree relatives of breast cancer (BC) patients present an increased risk for this malignancy. In particular, these individuals may have an increased susceptibility to cancer as a result of gene mutations present in parental germline cells. Tumors developing in these families are classified as hereditary cancers, and the most frequently involved predisposition genes include *BRCA1* and *BRCA2*. Overall, BRCA1/2-positive families present an increased incidence of breast, ovarian, prostate and pancreatic cancers [1,2,3,4]. Assessment of an individual’s risk for hereditary tumors is therefore based on a thorough evaluation of personal and family cancer history.

The identification of a mutation in *BRCA* genes plays a crucial role in the management of hereditary cancer prevention, diagnosis and treatment [2,5,6,7,8,9,10]. Nonetheless, because of the high costs associated with genetic analyses, especially in those countries where BRCA testing is offered by the national health service, BRCA testing has been restricted to BC patients having an a priori high risk of being carriers or candidates for approved targeted treatment strategies (i.e., PARP inhibitors [11]). In particular, according to the National Institute for Health and Care Excellence (NICE) in the UK, BRCA testing should be offered to BC patients with a probability of mutation ≥10% [12]. On the other hand, according to the recent update of NCCN guidelines [13], BRCA genetic testing is clinically recommended in individuals with a probability >5% based on prior probability models (e.g., Tyrer-Cuzik, BRCAPro etc). 

Several international oncology associations, such as ESMO, ASCO, NCCN etc., provide guidelines for BRCA testing based on the clinical–pathological characteristics of tumors and family cancer histories. On these grounds, in individuals potentially meeting established criteria for hereditary cancer syndrome, genetic testing is performed beginning with an appropriate examination of family history. Indeed, effective pretest counseling includes the development of an expanded pedigree that collects the health status of individuals diagnosed with cancer and first-, second- and third-degree relatives on both maternal and paternal sides. Nevertheless, several factors may limit the informativeness of the pedigree, such as small family size, a small number of individuals from the susceptible gender for sex-limited cancers, reduced penetrance, early deaths in family members, prophylactic surgeries that remove an organ due to subsequent cancer risk, adoptions, and inaccurate or incomplete information on family members [14,15]. Consequently, other factors should be considered during genetic counseling, including biology and age at diagnosis of the tumors developed by the counseled patient. In particular, the Italian Association of Medical Oncology (AIOM) guidelines include the personal history of triple-negative BC (TNBC) patients diagnosed ≤60 years and the personal history of early onset breast cancer (EOBC) patients diagnosed ≤35 years, regardless of family history [16], among the BRCA testing criteria. 

With the aim to evaluate the weight of clinical–pathological characteristics compared to tumor family histories, we evaluated the prevalence of BRCA germline mutations in an Italian cohort of TNBC and luminal-like EOBC patients without breast/ovarian cancer family histories.

## 2. Results

### 2.1. Triple-Negative Breast Cancer

Among 523 unselected TNBC patients diagnosed ≤60 years undergoing BRCA genetic testing at the MFCC, a total of 159 TNBC patients without BC and/or OC family histories were identified in our archives (Table 1). The prevalence of germline BRCA pathogenic or likely-pathogenic variants in the entire population of TNBC patients was 99/523 (18.9%), while the proportion among patients without a family history was 36/159 (22.6%). The BRCA detection rate was not significantly different between unselected TNBC patients and TNBC patients without a family history (*p* = 0.30). 

**Figure 1:**
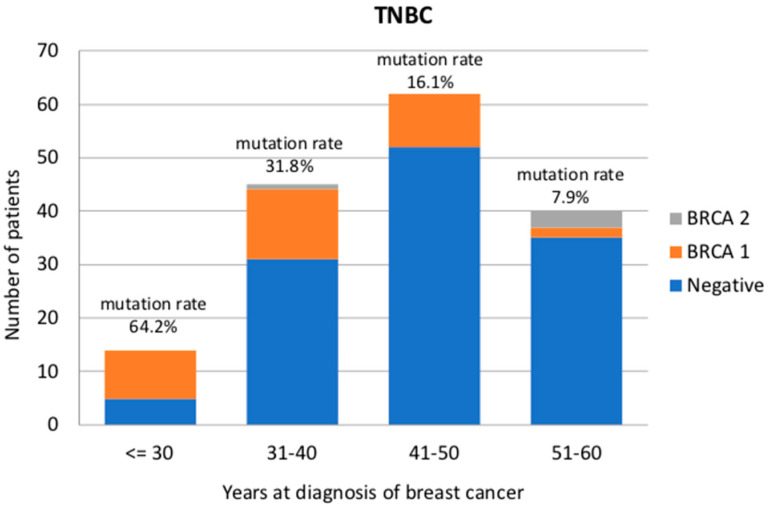
BRCA detection rate (%) in triple-negative breast cancer patients divided according to age at diagnosis (less than 30, 31–40, 41–50 and 51–60 years old).

Among TNBC patients without a family history, 34 patients presented a *BRCA1* pathogenic or likely-pathogenic variant (21.4%), whereas 2 patients were *BRCA2* carriers (1.2%). *BRCA1*-positive TNBC patients were diagnosed at a younger age (37 years) than noncarriers (44 years) or *BRCA2* carriers (45 years) (*p* < 0.001). Mutation prevalence in TNBC patients was 9/14 (64.2%) in the age group ≤30 years, 14/44 (31.8%) in 31–40 years, 10/62 (16.1%) in 41–50 years and 3/38 (7.9%) in 51–60 years (Figure 1). As expected, most of the TNBCs present a high proliferation rate and ductal histotype. Only one invasive lobular carcinoma was recorded and was categorized as BRCA1-associated. Moreover, three metaplastic carcinomas, two medullary, one sarcomatoid and one papillary tumor were diagnosed, and among these, one medullary and one papillary were diagnosed in BRCA1 carriers. Ten out of 159 patients presented ER and/or PR between 1% and 9%, and four of these (40%) were BRCA1 carriers. In particular, two of these patients were diagnosed at age 26 years old, one patient at 32 years and one patient at 56 years. No significant differences were observed between BRCA1 carriers and noncarriers in clinical and pathological characteristics such as ki-67 (*p* = 0.462), the presence of bilateral or second primary BC (*p* = 0.088), histotype (*p* = 0.301) or hormone receptor expression (*p* = 0.226).

Fourteen out of 159 patients (8.8%) presented a family history of pancreatic cancer. In particular, five women (two of which BRCA1 carriers) presented a first-degree relative affected by pancreatic cancer and eight women (two of which BRCA1 carriers) presented a second-degree relative with pancreatic cancer. Moreover, 22 out of 159 patients (13.8%) reported family history for prostate cancer. In detail, 6 women had a first-degree relative, 13 women (two of which BRCA1 carriers) had a second-degree relative, while 3 women (one BRCA1 carrier) presented two first or second-degree relatives affected by prostate cancers. In conclusion, the BRCA detection rate in TNBC patients with a family history of pancreatic cancer was 28.6%, whereas BRCA prevalence in women with both TNBC and a family history of prostate cancer was 13.6%. In both cases, the BRCA detection rate did not significantly differ from that of patients with pancreatic or prostate cancer family histories.

### 2.2. Early-Onset Luminal-like Breast Cancer

Among 646 unselected luminal-like EOBC patients undergoing BRCA genetic testing at the MFCC, a total of 109 luminal-like EOBC patients with no BC/OC family history were identified in our archives (Table 2). The *BRCA* detection rate among the entire population of luminal-like EOBC patients was 136/646 (21%), while the rate among EOBCs without a family history was 7/109 (6.4%). The BRCA detection rate in unselected luminal-like EOBC patients was significantly higher than in luminal-like EOBC patients without a family history (*p* = 0.0003). 

**Figure 2:**
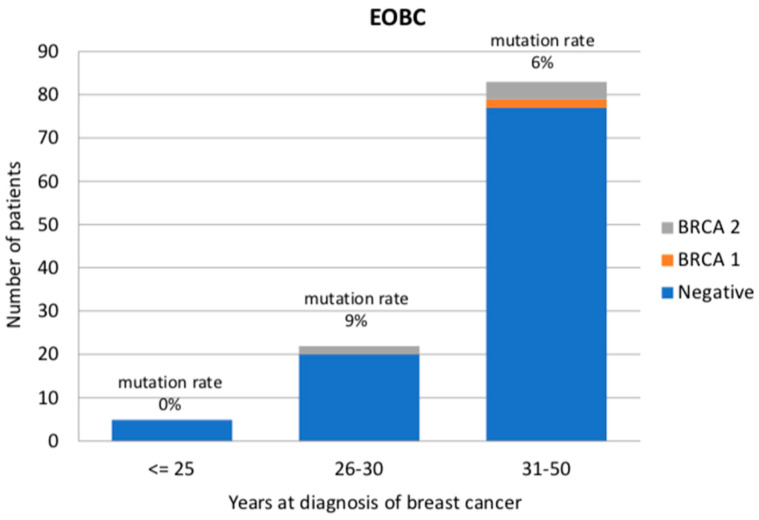
BRCA detection rate (%) in luminal-like early-onset breast cancer patients divided according to age at diagnosis (less than 25, 26–30 and 31–50 years old).

Among luminal-like EOBC patients without a family history, two patients presented a *BRCA1* pathogenic or likely-pathogenic variant (1.8%), whereas five patients were *BRCA2* carriers (4.6%). The mutation prevalence was 0/5 (0%) in the age group 0–25 years, 2/22 (9%) between 26 and 30 years, and 5/82 (6%) in the age group 31–35 years (Figure 2). Most patients were diagnosed with an invasive ductal carcinoma (in particular, 91.3% of the *BRCA*-negative patients and all *BRCA*-positive patients). One tubular, one papillary and one mucinous carcinoma were also diagnosed, all of them in patients who had tested negative for BRCA pathogenic or likely-pathogenic variants. No significant differences in age at diagnosis (*p* = 0.488), ki-67 (*p* = 0.920), presence of bilateral or second primary BC (*p* = 0.249), histotype (*p* = 1.00), PR (*p* = 0.105) or HER2 expression (*p* = 0.052) were observed.

Twelve out of 109 luminal-like EOBC patients (11%) presented a family history of pancreatic cancer. In particular, two women presented a first-degree relative, nine women (one BRCA2 carrier) presented a second-degree relative and one patient had one third-degree relative with pancreatic cancer. Moreover, 10 out of 109 patients (9.2%) reported a family history of prostate cancer. In detail, two women had a first-degree relative, six women (one BRCA2 carrier) had a second-degree relative, while two women presented two first or second-degree relatives affected by prostate cancers. In conclusion, the BRCA detection rate in luminal-like EOBC patients with a family history of pancreatic cancer was 8.3%, whereas BRCA prevalence in women with both luminal-like EOBC and a family history of prostate cancer was 10%. In both cases, the BRCA detection rate did not significantly differ from that of patients with pancreatic or prostate cancer family histories.

## 3. Discussion

According to the recommendations of all international oncology associations, BRCA genetic testing criteria are based on the clinical–pathological characteristics of personal tumor and family cancer histories. Pretest counseling is performed beginning with a thorough examination of family history. It is commonly thought that hereditary cancers should develop in the context of a family seriously affected by the same disease. Nevertheless, several factors may limit the informativeness of the pedigree, such as inaccurate or incomplete information on family members, while our patient’s cancer could be the first in the family. Indeed, a prospective study of 306 women diagnosed with breast cancer at <50 years of age, with no first or second-degree relatives with breast or ovarian cancer, showed that those individuals with a limited family history may have an underestimated probability of BRCA mutation, based on models relying exclusively on family history [17]. Moreover, mainstream test models characterized by few cancer-based criteria and provided directly by the cancer team instead of referring patients to the genetics department were demonstrated to efficiently deliver consistent and cost-effective BRCA testing [18]. Many authors highlight the need to increase our efforts in order to identify as many mutation carriers as possible, especially mutations in high-penetrance genes such as BRCA1 and BRCA2, possibly through national population testing programs [19]. Nevertheless, while we continue to wait for modelling, health economic and pilot implementation studies for population genetic testing, BRCA testing criteria are nowadays still needed, especially in countries where the test is offered by public health services.

Previous research helped to define the clinical–pathological characteristics of BRCA-related tumors. In detail, BRCA1-associated tumors are poorly differentiated infiltrating carcinomas, more frequently ER- and PR-negative and p53-positive [20,21]. On the other hand, BRCA2-associated BC tends to be of higher grade than sporadic age-matched controls [22]. Overall, BRCA-associated BCs are diagnosed at a young age and show a low frequency of HER2 expression [21,23]. On these grounds, international guidelines included the presence of young age at diagnosis and TN profile, regardless of family history, in the BRCA genetic testing criteria. Our study aimed to evaluate the rate of BRCA pathogenic or likely-pathogenic variants in patients selected according to the biology and age of diagnosis of their tumors, in the absence of cancer family history.

Our analyses indicated a high mutation rate (22.6%) in the overall population of TNBC patients diagnosed ≤60 years without BC and/or OC in their family history. In detail, BRCA testing was deemed cost-effective up to 50 years according to the NICE guidelines, while in the subgroup of patients diagnosed between 51 and 60 years, detection rate was 7.9%, and was still cost-effective according to the last NCCN guidelines. Interestingly, the BRCA detection rate was not significantly different between unselected TNBC patients and TNBC patients without a family history (*p* = 0.30). Overall, our results show some discrepancies from the previous literature. Indeed, according to the literature, 15–30% of unselected TNBCs had confirmed BRCA mutations [24,25,26]. Conversely, mutation prevalence decreased to 6–15% in patients without breast/ovarian cancer family histories [25,27]. The high BRCA mutation prevalence in our population could be explained by the fact that 37.1% of our patients were diagnosed ≤40 and 76.1% ≤ 50. Young age at diagnosis could therefore have increased the rate of BRCA detection compared to previous publications. Particularly, the high rate of young TNBC patients in our population could be explained by the fact that TNBC is more common in young patients and, until 2016, we only tested patients diagnosed ≤40 years. Finally, according to the literature, the BRCA1 patients in our study were diagnosed at a younger age than noncarriers.

Interestingly, despite the cut-off for hormone–receptor negativity defined by the ASCO–CAP guidelines [28], 40% of the patients with ER between 1% and 9% (ER low positive) were observed to be BRCA1-positive at genetic testing. Previous analyses have already highlighted that tumors with ER < 10% clinically behave as ER < 1% tumors [29]. Along with the results presented in this paper, these data indicate that, for clinical purposes, HER2-negative tumors with ER < 10% should be considered as TNBC. Furthermore, as reported in other analyses [19], 3 out of 34 (8.8%) BRCA1-related tumors presented a rarer non-ductal histotype (in our study, one medullary, one papillary and one lobular). Finally, no differences were observed between carriers and noncarriers in the proliferation rate and the presence of bilateral tumors or second primary BC. Since 2013, patients at our Family Cancer Clinic have been offered rapid genetic counseling and testing at BC diagnosis. This strategy was demonstrated to improve the rate of risk-reducing bilateral mastectomy at the time of BC surgery [30], enabling us to reduce the risk of contralateral tumors in BRCA carriers. Nevertheless, it is noteworthy that in the prospective POSH study, immediate bilateral mastectomy was not associated with improved survival. Hence, decisions about the timing of additional surgery aimed at reducing second primary cancer risks should still give priority to patient prognosis associated with the first malignancy and patient preferences [31].

In the second part of our analysis, we evaluated 109 patients diagnosed with luminal-like BC at ≤35 years without a family history of BC and/or OC. To our knowledge, this is the first study to evaluate the rate of BRCA mutation in luminal-like EOBC patients with no family history. This analysis is even more valuable in light of the EMBRACA trial results, in which talazoparib provided a significant PFS improvement over standard chemotherapy in patients with BRCA-related HER2-negative advanced breast cancer, at the same level in both TNBC and hormone-receptor-positive subtypes [32]. Overall, and in each age subgroup (≤25, 26–30 and 31–35), the BRCA detection rate was less than 10% (6.4%, 0%, 9% and 6%, respectively). Interestingly, all the patients diagnosed with EOBC under 26 years were observed to be negative at genetic testing, possibly underlining the need to evaluate other predisposing factors. In our population, the presence of BC and/or OC family history was demonstrated to significantly increase BRCA detection rate, which was 21% in unselected luminal-like EOBC patients and 24% in patients with family histories. No difference in proliferation rate was observed between carriers and noncarriers, contrary to what is reported in the literature [22]. Finally, as expected, most of hereditary luminal-like EOBCs were BRCA2-associated and HER2/neu-negative, and all of them were to be accounted for as ductal carcinoma. 

## 4. Materials and Methods

### 4.1. Study Population and Design

Since 1995, the Modena Family Cancer Clinic (MFCC) has offered genetic counseling to women with a personal or family history of BC and/or OC. In particular, healthy women with a family history are referred to the MFCC by their general practitioners or the radiologists that perform their population-based screening mammography. On the other hand, BC and OC patients are referred to the MFCC by their oncologists, radiologists, surgeons or gynecologists. During the first counseling visit at the clinic, women are classified in risk categories according to the Modena Criteria [33,34] and, more recently, using the Tyrer–Cuzick model [35], and are finally included in personalized surveillance programs. Moreover, individuals who meet the AIOM Criteria for genetic testing can undergo the BRCA test. Then, according to the result, they may access risk-reducing surgeries [30], chemoprevention studies [9] or more intensive surveillance programs [6,7]. During pretest counseling, family and personal histories of cancer are collected and a family pedigree is drawn including third-degree relatives on both maternal and paternal sides. Finally, after post-test counseling, a copy of all patient documents and reports are stored in the MFCC archive. 

For the purpose of our study, we retrospectively revised the 7690 family pedigrees stored in our archive and identified patients with TNBC diagnosed ≤60 years and luminal-like EOBC diagnosed ≤35 with neither breast nor ovarian family histories. The MFCC began to test all EOBC patients in 1998, whereas TNBC patients younger than 40 years have been tested since 2006. Only in 2016, the indication to genetic testing was extended to 60 years at TNBC diagnosis. 

ER, PR and HER2 expression was determined according to the national pathology guidelines, which closely adhere to international standards. For the clinical purpose of our study, triple negativity was defined as immunohistochemical staining of less than 10% of nuclei for both ER and PR, and an immunohistochemical result (DAKO score) of 0 or 1+ for HER2/neu or 2+ and DDISH/FISH-negative. Systematic evaluation of HER2 status became available in our institution only in 2006, when Trastuzumab was approved in the adjuvant setting. Hence, in our study, only triple-negative BC patients diagnosed from 2006 or with already performed retrospective HER2 evaluation were included. With regard to the luminal-like group, on the other hand, we included tumors with immunohistochemical staining of at least 10% of nuclei for ER. 

### 4.2. BRCA Testing Procedures

Before 2014, the genetic testing of BRCA1 and BRCA2 genes at our institution was carried out by direct Sanger sequencing, whilst it was performed using next-generation sequencing (NGS) after 2014. With both methods, the molecular test was performed on genomic DNA, isolated from fresh peripheral blood samples and encompassing the entire coding region as well as the adjacent intronic splice-site consensus sequences of BRCA genes. The NGS workflow benefited from the use of the Ion AmpliSeqTM technology, which was initially handled with a semi-automated procedure, and subsequently with a fully automated procedure for multiplex PCR-based library preparation and sequencing on the Ion Torrent platforms (Thermo Scientific). Sanger sequencing was routinely performed to validate candidate pathogenic or likely-pathogenic variants, as long as Multiplex Ligation Probe Amplification (MLPA, MRC-Holland) was carried out to detect copy number variations. NGS sequence alignment, base and variant calling relied on the Torrent Software Suite (Thermo Scientific) updated to the last available version at the time of sequencing, as well as the annotation process, which was also integrated with open source bioinformatics tools, customized and validated in the laboratory (Annovar [36] and Variant Effect Predictor, [37]) as described elsewhere [38,39]. For the purpose of this study, variants of unknown significance were considered as clinically negative results. Variants classified as pathogenic or likely-pathogenic at the time of diagnosis were re-evaluated at the time of manuscript writing according to the ACMG criteria (American College of Medical Genetics and Genomics) [40] for possible classification updating. All the variants classified as pathogenic or likely-pathogenic were recognized as such. 

### 4.3. Statistical Analysis

Mutation prevalence and clinical–pathological characteristics were evaluated for each subgroup of patients. Patient characteristics and the distribution of each parameter across subgroups were reported as absolute and percentage frequencies. A comparison between groups was made by means of Fischer’s exact test. Statistical analyses were conducted using IBM SPSS Statistics for Windows Version 23.0 (IBM Corporation, Armonk, NY, USA). P-values < 0.05 were considered significant.

## 5. Conclusions

According to the last NCCN Guidelines, the results of our study confirm the recommendation to test for BRCA genes in TNBC patients ≤ 60 years, regardless of family history, tumor histotype and by using immunohistochemical staining of less than 10% of nuclei for both ER and/PR as a cut-off. In luminal-like EOBC patients with no BC and/or OC family history, on the other hand, a lower overall BRCA detection rate was observed of >5%, suggesting a role for testing other predisposing genes along with BRCA1 and BRCA2 in this subset of patients. Finally, in both TNBC and luminal-like EOBC groups, the presence of pancreatic or prostate cancers in family histories did not significantly modify the BRCA detection rate. 

## Figures and Tables

**Table 1:** Characteristics of triple-negative breast cancer patients.

TNBC	Negative	BRCA1	BRCA2	*p*-Value	
**Number of patients** (159)	123	34	2	*	**
**Mean age at diagnosis (y)**	44.78 (24–59) SD 9.07	36.97 (26–54) SD 8.74	45.00 (39–51) SD 8.48	0.004	<0.001
**Age group (y)**				<0.001	<0.001
**≤30**	5 (4.0%)	9 (26.5%)	0		
**31–40**	31 (25.2%)	13 (38.3%)	1 (50%)		
**41–50**	52 (42.3%)	10 (29.4%)	0		
**51–60**	35 (28.5%)	2 (5.9%)	1 (50%)		
**Ki 67 (%)**				0.508	0.462
≤20	11 (10.1%)	1 (3.3%)	0		
>20	98 (89.9%)	29 (96.7%)	1 (100%)		
unknown	14	4	1		
**Bilaterality**				0.193	0.088
Yes	5 (4.2%)	4 (12.9%)	0		
No	114 (95.7%)	27 (87.1%)	2 (100%)		
unknown	4	3	0		
**Histotype**				0.466	0.301
ductal	103 (95.4%)	28 (90.3%)	2 (100%)		
lobular	0	1 (3.2%)	0		
others	5 (4.6%)	2 (6.5%)	0		
unknown	15	3	0		
**RO**				0.321	0.226
negative	116 (95.1%)	30 (88.2%)	2 (100%)		
1–9 %	6 (4.9%)	4 (11.8%)	0		
unknown	1	0	0		

* Comparison Negative vs. BRCA1 vs. BRCA2; ** Comparison Negative vs BRCA1.

**Table 2:** Characteristics of luminal-like early onset breast cancer patients.

EOBC	Negative	BRCA1	BRCA2	*p*-Value
**Number of patients (109)**	102	2	5	*
**Mean age at diagnosis (y)**	31.95 (23–35) SD 2.85	34.0 (33–35) SD 2.93	30.6 (27–33) SD 2.89	0.488
**Age group (y)**				0.688
**≤25**	5 (4.9%)	0	0	
**26–30**	20 (19.6%)	0	2 (40%)	
**31–35**	77 (75.5%)	2 (100%)	3 (60%)	
**Ki 67 (%)**				0.920
**≤20**	35 (44.9%)	0	2 (50%)	
**>20**	43 (55.1%)	1 (100%)	2 (50%)	
**unknown**	24	1	1	
**Bilaterality**				0.249
**Yes**	5 (5.3%)	1 (50%)	0	
**No**	90 (94.7%)	1 (50%)	5 (100%)	
**unknown**	7	0	0	
**Histotype**				1.00
**ductal**	74 (91.3%)	2 (100%)	4 (100%)	
**lobular**	3 (3.7%)	0	0	
**others**	4 (4.9%)	0	0	
**unknown**	21	0	1	
**PR**				0.105
**≤20**	29 (33.7%)	0	2 (40%)	
**>20**	57 (66.3%)	0	3 (60%)	
**unknown**	23	2	0	
**HER2**				0.052
**negative**	54 (69.2%)	0	5 (100%)	
**positive**	24 (30.8%)	0	0	
**unknown**	24	2	0	

* Comparison Negative vs. BRCA1 vs. BRCA2.

## References

[1] Toss A., Molinaro E., Sammarini M., Del Savio M.C., Cortesi L., Facchinetti F., Grandi G. (2019). Hereditary ovarian cancers: State of the art. Minerva Med..

[2] Toss A., Venturelli M., Molinaro E., Pipitone S., Barbieri E., Marchi I., Tenedini E., Artuso L., Castellano S., Marino M. (2019). Hereditary Pancreatic Cancer: A Retrospective Single-Center Study of 5143 Italian Families with History of BRCA-Related Malignancies. Cancers.

[3] Lecarpentier J., Silvestri V., Kuchenbaecker K.B., Barrowdale D., Dennis J., McGuffog L., Soucy P., Leslie G., Rizzolo P., Navazio A.S. (2017). Prediction of Breast and Prostate Cancer Risks in Male BRCA1 and BRCA2 Mutation Carriers Using Polygenic Risk Scores. J. Clin. Oncol..

[4] Kuchenbaecker K.B., Hopper J.L., Barnes D.R., Phillips K.A., Mooij T.M., Roos-Blom M.J., Jervis S., van Leeuwen F.E., Milne R.L., Andrieu N. (2017). Risks of Breast, Ovarian, and Contralateral Breast Cancer for BRCA1 and BRCA2 Mutation Carriers. JAMA.

[5] Grandi G., Sammarini M., Del Savio M.C., Toss A., Facchinetti F. (2019). Combined hormonal contraceptives in *BRCA* gene mutation carriers: Why not?. Eur. J. Contracept. Reprod Health Care.

[6] Cortesi L., Canossi B., Battista R., Pecchi A., Drago A., Dal Molin C., Toss A., De Matteis E., Marchi I., Torricelli P. (2019). Breast ultrasonography (BU) in the screening protocol for women at hereditary-familial risk of breast cancer: Has the time come to rethink the role of BU according to different risk categories?. Int. J. Cancer.

[7] Cortesi L., De Matteis E., Toss A., Marchi I., Medici V., Contu G., Xholli A., Grandi G., Cagnacci A., Federico M. (2017). Evaluation of Transvaginal Ultrasound plus CA-125, Measurement and Prophylactic Salpingo-Oophorectomy in Women at Different Risk Levels of Ovarian Cancer: The Modena Study Group Cohort Study. Oncology.

[8] Toss A., Grandi G., Cagnacci A., Marcheselli L., Pavesi S., De Matteis E., Razzaboni E., Tomasello C., Cascinu S., Cortesi L. (2017). The impact of reproductive life on breast cancer risk in women with family history or BRCA mutation. Oncotarget.

[9] Razzaboni E., Toss A., Cortesi L., Marchi I., Sebastiani F., De Matteis E., Federico M. (2013). Acceptability and adherence in a chemoprevention trial among women at increased risk for breast cancer attending the Modena Familial Breast and Ovarian Cancer Center (Italy). Breast J..

[10] Patel V.L., Busch E.L., Friebel T.M., Cronin A., Leslie G., McGuffog L., Adlard J., Agata S., Agnarsson B.A., Ahmed M. (2020). Association of Genomic Domains in BRCA1 and BRCA2 with Prostate Cancer Risk and Aggressiveness. Cancer Res..

[11] Cortesi L., Toss A., Cucinotto I. (2018). PARP Inhibitors for the Treatment of Ovarian Cancer. Curr. Cancer Drug Targets.

[12] NICE Guideline. Familial Breast Cancer: Classification, Care and Managing Breast Cancer and Related Risks in People with a Family History of Breast Cancer. www.nice.org.uk/guidance/cg164.

[13] NCCN Clinical Practice Guidelines in Oncology. Genetic/Familial High-Risk Assessment: Breast, Ovarian, and Pancreatic Version 1.2020. https://www.nccn.org/professionals/physician_gls/pdf/genetics_bop.pdf.

[14] Berliner J.L., Fay A.M. (2007). Practice Issues Subcommittee of the National Society of Genetic Counselors’ Familial Cancer Risk Counseling Special Interest Group. Risk assessment and genetic counseling for hereditary breast and ovarian cancer: Recommendations of the National Society of Genetic Counselors. J. Genet. Couns..

[15] Calzone K.A., Soballe P.W. (2008). Genetic testing for cancer susceptibility. Surg. Clin. N. Am..

[16] AIOM Guidelines 2019. Neoplasie Della Mammella. https://www.aiom.it/wp-content/uploads/2019/10/2019_LG_AIOM_Mammella.pdf.

[17] Weitzel J.N., Lagos V.I., Cullinane C.A., Gambol P.J., Culver J.O., Blazer K.R., Palomares M.R., Lowstuter K.J., MacDonald D.J. (2007). Limited family structure and BRCA gene mutation status in single cases of breast cancer. JAMA.

[18] Kemp Z., Turnbull A., Yost S., Seal S., Mahamdallie S., Poyastro-Pearson E., Warren-Perry M., Eccleston A., Tan M.M., Teo S.H. (2019). Evaluation of Cancer-Based Criteria for Use in Mainstream BRCA1 and BRCA2 Genetic Testing in Patients With Breast Cancer. JAMA Netw. Open.

[19] Turnbull C., Sud A., Houlston R.S. (2018). Cancer genetics, precision prevention and a call to action. Nat. Genet..

[20] Turchetti D., Cortesi L., Federico M., Bertoni C., Mangone L., Ferrari S., Silingardi V. (2000). BRCA1 mutations and clinicopathological features in a sample of Italian women with early-onset breast cancer. Eur. J. Cancer.

[21] Honrado E., Benítez J., Palacios J. (2006). Histopathology of BRCA1- and BRCA2-associated breast cancer. Crit. Rev. Oncol. Hematol..

[22] Lakhani S.R., Gusterson B.A., Jacquemier J., Sloane J.P., Anderson T.J., van de Vijver M.J., Venter D., Freeman A., Antoniou A., McGuffog L. (2000). The pathology of familial breast cancer: Histological features of cancers in families not attributable to mutations in BRCA1 or BRCA2. Clin. Cancer Res..

[23] Litton J.K., Ready K., Chen H., Gutierrez-Barrera A., Etzel C.J., Meric-Bernstam F., Gonzalez-Angulo A.M., Le-Petross H., Lu K., Hortobagyi G.N. (2012). Earlier age of onset of BRCA mutation-related cancers in subsequent generations. Cancer.

[24] Greenup R., Buchanan A., Lorizio W., Rhoads K., Chan S., Leedom T., King R., McLennan J., Crawford B., Kelly Marcom P. (2013). Prevalence of BRCA mutations among women with triple-negative breast cancer (TNBC) in a genetic counseling cohort. Ann. Surg. Oncol..

[25] Sharma P., Klemp J.R., Kimler B.F., Mahnken J.D., Geier L.J., Khan Q.J., Elia M., Connor C.S., McGinness M.K., Mammen J.M. (2014). Germline BRCA mutation evaluation in a prospective triple-negative breast cancer registry: Implications for hereditary breast and/or ovarian cancer syndrome testing. Breast Cancer Res. Treat..

[26] Muendlein A., Rohde B.H., Gasser K., Haid A., Rauch S., Kinz E., Drexel H., Hofmann W., Schindler V., Kapoor R. (2015). Evaluation of BRCA1/2 mutational status among German and Austrian women with triple-negative breast cancer. J. Cancer Res. Clin. Oncol..

[27] Engel C., Rhiem K., Hahnen E., Loibl S., Weber K.E., Seiler S., Zachariae S., Hauke J., Wappenschmidt B., Waha A. (2018). Prevalence of pathogenic BRCA1/2 germline mutations among 802 women with unilateral triple-negative breast cancer without family cancer history. BMC Cancer.

[28] Allison K.H., Hammond M.E.H., Dowsett M., McKernin S.E., Carey L.A., Fitzgibbons P.L., Hayes D.F., Lakhani S.R., Chavez-MacGregor M., Perlmutter J. (2020). Estrogen and Progesterone Receptor Testing in Breast Cancer: ASCO/CAP Guideline Update. J. Clin. Oncol..

[29] Fujii T., Kogawa T., Dong W., Sahin A.A., Moulder S., Litton J.K., Tripathy D., Iwamoto T., Hunt K.K., Pusztai L. (2017). Revisiting the definition of estrogen receptor positivity in HER2-negative primary breast cancer. Ann. Oncol..

[30] Cortesi L., Razzaboni E., Toss A., De Matteis E., Marchi I., Medici V., Tazzioli G., Andreotti A., De Santis G., Pignatti M. (2014). Rapid genetic counselling and testing in newly diagnosed breast cancer is associated with high rate of risk-reducing mastectomy in BRCA1/2-positive Italian women. Ann. Oncol..

[31] Copson E.R., Maishman T.C., Tapper W.J., Cutress R.I., Greville-Heygate S., Altman D.G., Eccles B., Gerty S., Durcan L.T., Jones L. (2018). Germline BRCA mutation and outcome in young-onset breast cancer (POSH): A prospective cohort study. Lancet Oncol..

[32] Litton J.K., Rugo H.S., Ettl J., Hurvitz S.A., Gonçalves A., Lee K.H., Fehrenbacher L., Yerushalmi R., Mina L.A., Martin M. (2018). Talazoparib in Patients with Advanced Breast Cancer and a Germline BRCA Mutation. N. Engl. J. Med..

[33] Federico M., Maiorana A., Mangone L., Turchetti D., Canossi B., Romagnoli R., Silingardi V. (1999). Identification of families with hereditary breast and ovarian cancer for clinical and mammographic surveillance: The Modena Study Group proposal. Breast Cancer Res. Treat..

[34] Cortesi L., Turchetti D., Marchi I., Fracca A., Canossi B., Rachele B., Silvia R., Rita P.A., Pietro T., Massimo F. (2006). Breast cancer screening in women at increased risk according to different family histories: An update of the Modena Study Group experience. BMC Cancer.

[35] Tyrer J., Duffy S.W., Cuzick J. (2004). A breast cancer prediction model incorporating familial and personal risk factors. Stat. Med..

[36] Wang K., Li M., Hakonarson H. (2010). ANNOVAR: Functional annotation of genetic variants from high-throughput sequencing data. Nucleic Acids Res..

[37] McLaren W., Pritchard B., Rios D., Chen Y., Flicek P., Cunningham F. (2010). Deriving the consequences of genomic variants with the Ensembl API and SNP Effect Predictor. Bioinformatics.

[38] Artusi V., Chiesi L., Bernardis I., Tenedini E., Artuso L., Cavallini G.M., Percesepe A., Marigo V., Tagliafico E. (2015). A Next Generation Sequencing amplicon-based strategy to explore inherited Retinal Degeneration complexity. Eur. J. Hum. Gen..

[39] Tenedini E., Artuso L., Bernardis I., Artusi V., Percesepe A., De Rosa L., Contini R., Manfredini R., Pellacani G., Pagani J. (2015). Amplicon-based next-generation sequencing: An effective approach for the molecular diagnosis of epidermolysis bullosa. Br. J. Dermatol..

[40] Richards S., Aziz N., Bale S., Bick D., Das S., Gastier-Foster J., Grody W.W., Hegde M., Lyon E., Spector E. (2015). Standards and guidelines for the interpretation of sequence variants: A joint consensus recommendation of the American College of Medical Genetics and Genomics and the Association for Molecular Pathology. Genet. Med..

